# Framing Mental Health Within Digital Games: An Exploratory Case Study of Hellblade

**DOI:** 10.2196/12432

**Published:** 2019-04-18

**Authors:** Joseph Fordham, Christopher Ball

**Affiliations:** 1 Department of Media and Information Michigan State University East Lansing, MI United States; 2 Department of Journalism University of Illinois at Urbana-Champaign Urbana-Champaign, IL United States

**Keywords:** video games, electronic gaming, psychosis, stigma

## Abstract

**Background:**

Researchers and therapists have increasingly turned to digital games for new forms of treatments and interventions for people suffering from a variety of mental health issues. Yet, the depiction of mental illness within digital games typically promotes stigmatized versions of those with mental health concerns. Recently, more games have attempted to implement more realistic and respectful depictions of mental health conditions.

**Objective:**

This paper presents an exploratory analysis of a contemporary game that has the potential to change the way researchers, practitioners, and game designers approach topics of mental health within the context of gaming.

**Methods:**

A case study of Hellblade: Senua's Sacrifice was conducted using frame analysis to show how key design choices for this game present the potential for new ways of approaching games and mental health.

**Results:**

A case study of Hellblade’s development shows how research-informed collaborative design with mental health practitioners, scientists, and individuals with mental health problems can lead to a realistic depiction of mental illness in games. Furthermore, the use of frame analysis demonstrates how to harness narrative, mechanics, and technology to create embodied experiences of mental health, which has the potential to promote empathetic understanding.

**Conclusions:**

This paper highlights an exemplary case of collaborative commercial game design for entertainment purposes in relation to mental health. Understanding the success of Hellblade's depiction of psychosis can improve serious games research and design. Further research must continue to provide deeper analysis of not only games that depict mental illness, but also the design process behind them.

## Introduction

### Background

The growth of digital games in terms of both popularity and technology has pushed games into various roles beyond simply entertainment. Digital games and game design practices have been applied to fitness [1], job training [2], education [3], and even therapeutic practices [4]. Not only are games being applied to various roles, but designers are also approaching more serious topics such as humanitarian crises [5], the realities of war [6], and mental health [7]. The integration of *serious* topics within digital games brings with it debates regarding the acceptability of “playing” with such serious subject matter [8]. These issues, particularly notions of trivialization or stigmatization, are common among larger commercial games created primarily for profit. Researchers and smaller independent game designers frequently create *serious games* that are typically smaller games or game-like experiences whose primary purpose is not simply entertainment.

Hellblade: Senua’s Sacrifice, hereby also referred to as Hellblade, is a digital game developed and independently published by Ninja Theory, which is a British game development studio based out of Cambridge, England [9]. In Hellblade, players take on the role of Senua, a female Pict warrior on a quest that quite literally tests her sanity. Hellblade’s presentation of Senua’s psychosis provides interesting insights into how games can depict challenging, controversial topics [10,11]. Hellblade is significant because it is one of only a very few commercial video games to ever tackle the serious topic of mental illness [12] and perhaps the most successful, returning a profit in sales after only 3 months [13]. As a result, Hellblade is a game that could contribute to numerous conversations across various fields, such as psychology and game studies.

This paper analyzes Hellblade in two ways. First, Hellblade is used as a case study in which a formal analysis was conducted. This formal analysis was used to identify the components, actions, and goals present in Hellblade [14]. Secondly, this study turns to the use of frame analysis in order to better understand the design and development decisions used by Ninja Theory to depict symptoms of psychosis and how future games can emulate Hellblade’s success. Specifically, we examine how various frames and design choices were used to create a compelling experience related to mental illness while alleviating concerns of trivializing such a serious topic. The results of this study should help us better understand Hellblade’s depiction of mental illness but also the collaborative design process behind its creation.

### Games and Mental Health

With regard to games and mental health, researchers and therapists have increasingly turned to digital games for new forms of treatments and interventions for those dealing with mental health issues. Research shows that games appear to be well-suited for promoting mental health awareness and possibly as interventions [15,16]. Games can increase the impact of mental health interventions due, in part, to their broad appeal, accessibility, and ability to keep participants engaged [17]. In fact, a number of commercial games have been adopted and tested as unique forms of therapy [18,19]. Thorens et al [20] argue that the naturalistic use (ie, outside of traditional interventions) of digital games may be particularly helpful given game design practices that motivate players to continue to play, such as online, social play, and reward schemes. This is noteworthy given the number of people who play digital games and, by extension, the potential to reach those who face mental health issues but do not seek out or receive treatment [17]. As Goodman et al [21] point out, the nature of online gaming communities provides interesting possibilities in terms of possible health interventions and support.

Unfortunately, the combination of games and mental health comes with several potential issues. Most notably, there is a lack of cooperation between game designers and mental health specialists, as well as the exclusion of individuals dealing with these disorders, which leads to a lack of games that are both beneficial and engaging [22]. This is evident in publications from both mental health and game design researchers attempting to prescribe a formula for working across these disciplines successfully [22-24]. Mental health researchers and practitioners attempting to make use of serious game-based interventions often deal with the lack of an engaging, well-constructed gameplay experience [17]. Similarly, serious games and apps crafted without oversight by mental health professionals risk both ethical issues and possible conflict with therapeutic goals [25]. Meanwhile, commercial games depicting various forms of mental illness tend to be crafted with problematic, stigmatized versions of individuals dealing with mental health issues in such a way that shows little-to-no understanding of the actual disorders they claim to depict [16].

The inclusion of mental health disorders within digital games has often been viewed as troublesome. This is most notable in relation to horror-themed games, which often rely on heavily stigmatized versions of the “mentally ill” as prone to violence and mental health hospitals as dark, haunted asylums [11,26]. Sendler [27] argues that mental illness has increasingly been presented by the media because of society’s growing desensitization and fascination toward these disorders. Many media forms, particularly games, turn to a base notion of “insanity” to explain seemingly inexplicable acts of violence perpetrated by the game’s villains [28]. Games such as Outlast (Red Barrels), Manhunt (Rockstar Games), and The Evil Within (Bethesda Softworks) include harmful, stereotypical depictions of these characters, including murderous patients or abusive caretakers [16,29]. These troublesome portrayals of individuals experiencing forms of mental illness promote fear and stigma toward people dealing with these issues while perhaps also promoting forms of self-stigmatization, which may inhibit people dealing with mental health concerns from attempting to seek help and support [26].

This is not to say that all games that choose to depict aspects of mental illnesses are as problematic. However, video games that tackle serious topics in an accurate and empathetic way are not only rare, but are often much smaller, independently developed games that struggle to find a wide audience amidst larger commercial releases. Games such as Depression Quest (Zoe Quinn) and Pry (Tender Claws) have received praise for allowing players to navigate narratives related to mental health issues [7,29-31]. Depression Quest is a text-driven game that asks the player to take on the role of a person dealing with depression. In a similar vein to a choose-your-own-adventure book, players make decisions based on various prompts similar to events experienced in daily life. Over time, players find that certain choices are visible but not accessible to them, alluding to struggles with anxiety, fatigue, and pessimism that are common symptoms for those dealing with depression. Pry acts similarly to an interactive or visual novel. Pry asks the player to explore the mind of a soldier dealing with posttraumatic stress disorder. Players are given access to various pictures and text and are given the ability to interact with these various prompts to learn more about their character’s memories, all while attempting to balance the line between the real world and the soldier’s subconscious. Smaller, independent games such as Depression Quest and Pry are largely text-driven and usually developed by individuals or, at best, fairly small teams of designers. These serious-topic focused *indie* games are typically meant to share a message rather than simply be “played” in a traditional entertainment sense; they also receive little commercial consideration. While such games are commendable for tackling important topics in a more careful and accurate way, they often suffer from mixed reviews and relatively low sales.

Larger commercial games that call upon various forms of psychological disorders are typically met with a great deal of skepticism. As mentioned earlier, the notion of “playing” these types of games typically brings accusations of trivializing or exploiting topics that are perceived to require a more reverent approach. Chapman and Linderoth [8] refer to this notion of certain themes being deemed inappropriate for inclusion within the context of games as the “limits of play.” In their work on Nazism in games, Chapman and Linderoth [8] argue that “the tension between the inevitable un-seriousness of games and sensitive themes is only likely to survive if an overarching frame is established.” In that vein, it is essential that researchers and designers attempt to examine not only the portrayal of mental illness and mental health in video games, but also the design practices that encourage an increased empathetic understanding of people with mental health problems [32]. Therefore, we need a better understanding of the process by which we can develop games that not only address issues related to mental health, but also have the potential to reach a wider audience. In this exploratory study, we attempt to address both of these needs by conducting a formal analysis of one particular game, Hellblade: Senua’s Sacrifice [9].

### Hellblade: A Collaborative Design Template

This paper presents an exploratory case study of a single game, Hellblade, from its development to reception, as a lens through which to understand how games can depict mental illness in a respectful and nonstigmatizing fashion. Unlike most serious games or the previously mentioned Depression Quest and Pry, Hellblade was created by an established AAA game design studio, Ninja Theory, as a fully developed commercial title that was released across multiple gaming platforms. In general, AAA games are considered to be of a high quality, created by relatively large studios and with relatively large budgets [33]. Despite their game’s commercial aspirations, the designers at Ninja Theory devoted themselves to creating a respectful depiction of psychosis, going so far as to develop the game independent of publisher support and funding. Hellblade was created and published solely by Ninja Theory to ensure the game they envisioned was not subject to outside pressures, a process they referred to as “the independent AAA proposition” [34]. This proposition resulted in one of the most noteworthy and best-selling digital games to focus on serious subject matter such as mental health [13].

This paper argues that Ninja Theory’s willingness to collaborate with mental health professionals, researchers, and individuals actively dealing with mental health disorders resulted in design choices that allow Hellblade to present a respectful and engaging depiction of psychosis. Specifically, using frame analysis [35], we illustrate how elements of Hellblade’s design allowed Ninja Theory to successfully craft a game built upon notions of psychosis. This analysis is reinforced through development diaries and press interviews given throughout the game’s creation. Through a formal analysis of Hellblade and further frame analysis of Ninja Theory’s developmental process and design choices, this paper provides insights into how the cooperation of designers, researchers, practitioners, and individuals with mental health problems can allow games to depict challenging, controversial topics successfully.

## Methods

### Case Study

This paper presents a case study of Hellblade: Senua’s Sacrifice (PC). In order to accomplish this, a formal analysis of the game was performed by both researchers (JF and CB) through sessions of both active gameplay and the observation of recorded gameplay by the authors [14]. Formal analysis with relation to game studies, also referred to as aesthetic analysis [36] or simply game analysis [37], is an examination based primarily upon the “playing of a game and attempting to understand how the game system works” [14]. This analysis relies on the base categories of “objects, interface, and interactions” put forth by Consalvo and Dutton [37]. The study of a single game and its components could easily fill an entire book, yet, as Aarseth [36] argues, this form of inquiry requires the researcher to focus and pursue the aspects that make a game noteworthy.

**Figure 1:**
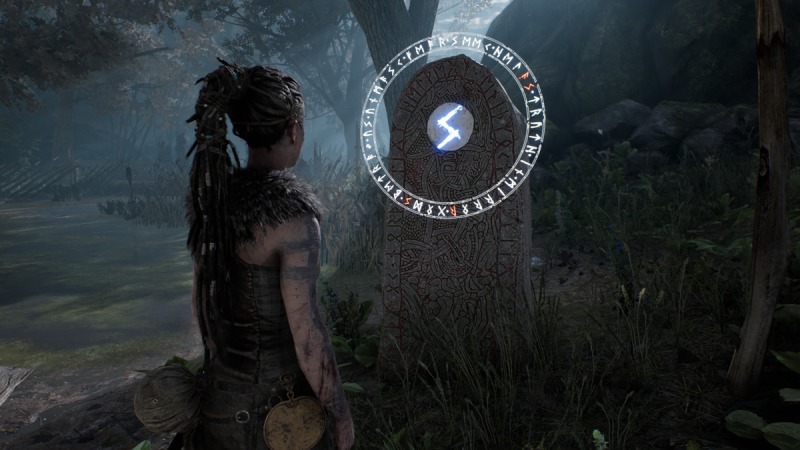
Example of Norse lorestones in the game Hellblade (screenshot).

In terms of methodology, this form of analysis must rely primarily upon the act of playing a particular game. Lankoski and Bjork [14] point out that “game dynamics are more easily understood in actual gameplay” with an eye to “the elements that constitute the parts of the work and the role of each element in the composition as a whole.” In that sense, gameplay logs [37] were kept by researchers throughout multiple play and viewing sessions. These logs noted objects within the game space (eg, lorestones), the presence or lack of an interface (eg, no on-screen health bar, but darkening of the screen to denote danger), and interactions possible within the game (eg, activating and listening to lorestones or discovering glyphs) (see Figure 1). These initial logs provided a base understanding of Hellblade’s gameplay, structure, and the world it presents.

While Aarseth [36] argues that “informed game scholarship must involve play,” he also notes that this should be reinforced through secondary sources such as interviewing players or developers. The initial gameplay logs were further informed by a 25-minute documentary created by Ninja Theory and included with every copy of Hellblade [38]. This documentary focused primarily on the role of psychosis within Hellblade’s design and on how these decisions were informed by the researchers and individuals dealing with these symptoms whom Ninja Theory included throughout the development process. For further data on Hellblade’s development, 30 developer diaries, three question-and-answer sessions held by the lead developers, and a masterclass session given by creative director Tameem Antoniades for the British Academy of Film and Television Arts (BAFTA) were accessed via the official website for Hellblade as well as through Ninja Theory’s YouTube portal [9,39].

Ninja Theory’s developer diaries span the length of Hellblade’s development. The first, published in August 2014, details the creation of the game’s announcement trailer, which had been released 2 weeks prior [40]; the final diary (#30), detailing Hellblade’s launch and early reception, was posted 3 months after the game’s release in 2017 [41]. Each diary focuses on a singular aspect of the game’s design: from art and narrative inspirations and character design to the inclusion of mental health researchers and practitioners throughout the development and playtesting process. These diaries were made publicly available on both Ninja Theory’s YouTube channel and the official website for Hellblade. These diaries were led by the game’s creative director Tameem Antoniades and lead producer Dominic Matthews, although other designers, character actors, and mental health professionals were also included, depending on each video’s topic.

These developer insights helped provide further context for the initial gameplay logs as well as new insight into Hellblade’s design. A final playthrough and observation of Hellblade was performed that was particularly focused on identifying and further understanding how game design elements contributed to the game’s depiction of Senua’s psychosis. Through this process, four main themes were chosen to discuss how Ninja Theory framed their depiction of psychosis within Hellblade: narrative and storytelling, in-game mechanics, platform and technological affordances, and an open dedication to collaboration and care.

### Frame Analysis

Frame analysis has become an increasingly common analytical tool for game scholars [42,43]. Goffman’s notion of a *frame* relates to the meaning we attribute to a situation. These frames are largely based upon social or cultural understandings and how we use them to interpret meaning. For instance, the act of playing a game is typically viewed as not serious as well as separate from real life. Thus, when playing violent video games, most players have no qualms performing criminal activities they would likely never perform in the real world. This notion of a game frame [44], or what Chapman and Linderoth [8] refer to as the ludic frame, typically involves the redefinition of an object or activity based on its inclusion within a game. This ludic frame suggests a more playful and less serious approach to a given topic through a process Goffman refers to as *keying* [35].

Keying is the shift in the meaning of an activity that occurs within a given frame. For example, the ways in which a player engages with a game will change dependent upon the situation and meaning attributed to the play session. Professional athletes may approach the act of playing a game under the premise of training rather than simply leisure enjoyment. This additional frame of *professional sport* adds new meaning to the act of playing, a process known as *upkeying* [26,45]. In contrast, the act of *downkeying* involves the removal of additional frames toward a traditional understanding of a given situation.

With relation to games and depictions of mental illness, it is important to consider the steps developers are taking in order to signal certain meanings to players. Chapman and Linderoth [8] discuss this negotiation between what is and is not deemed appropriate for digital games in their study of the depiction of Nazism in games. In particular, they note that “ludic frames seem to have an intrinsically trivializing property,” [8] yet concerns over the depiction of controversial topics in games can sometimes be alleviated by developers positioning their work within frames of artistic expression, education, or a documentary-like devotion to accuracy. These various forms of framing content allow for an upkeying of the game activity to a more serious understanding of a game’s content.

Commercial games depicting aspects of mental health conditions are often criticized for their stigmatic portrayal of these disorders. While numerous indie games approach topics of mental health in creative and respectful ways, these larger commercial products are promoted and distributed by large-scale publishers and typically reach much larger audiences [16]. Depictions of fear and violence attributed to mental health disorders within these larger games promote the same stigmas and stereotypes commonly seen in television and film [11]. In order to create a game that may help alleviate these stigmas and present an engaging representation of psychosis, Ninja Theory relied on design choices that help to frame Hellblade as a respectful interpretation of these conditions, upkeying the play experience to a more serious, meaningful tone. By identifying the ways in which Hellblade presents its depiction of psychosis to the player, this paper helps illustrate methods with which game designers can better approach more serious topics and why these more positive depictions of stigmatized populations are important areas for games to explore.

## Results

### A Narrative of Psychosis

Hellblade approaches the topic of mental health in ways that reflect both its commercial and serious aspirations. Commercial games, typically focused on violence or horror, tend to depict those with mental health issues as antagonists. There is usually little exploration or explanation as to their situation, relying on stereotyped notions of mental health as a generic explanation for dangerous, violent characters within a larger, fantasy narrative [11]. Serious and independent games that deal with mental health issues typically devote the entire game to their depiction of these disorders. For example, the previously mentioned Depression Quest immediately places the player in the role of someone dealing with depression and task them with making decisions about this character’s everyday life. Hellblade attempts to combine these approaches: placing players in the role of Senua and attempting to present a faithful representation of psychosis, while presenting players with a larger narrative and world to explore.

**Figure 2:**
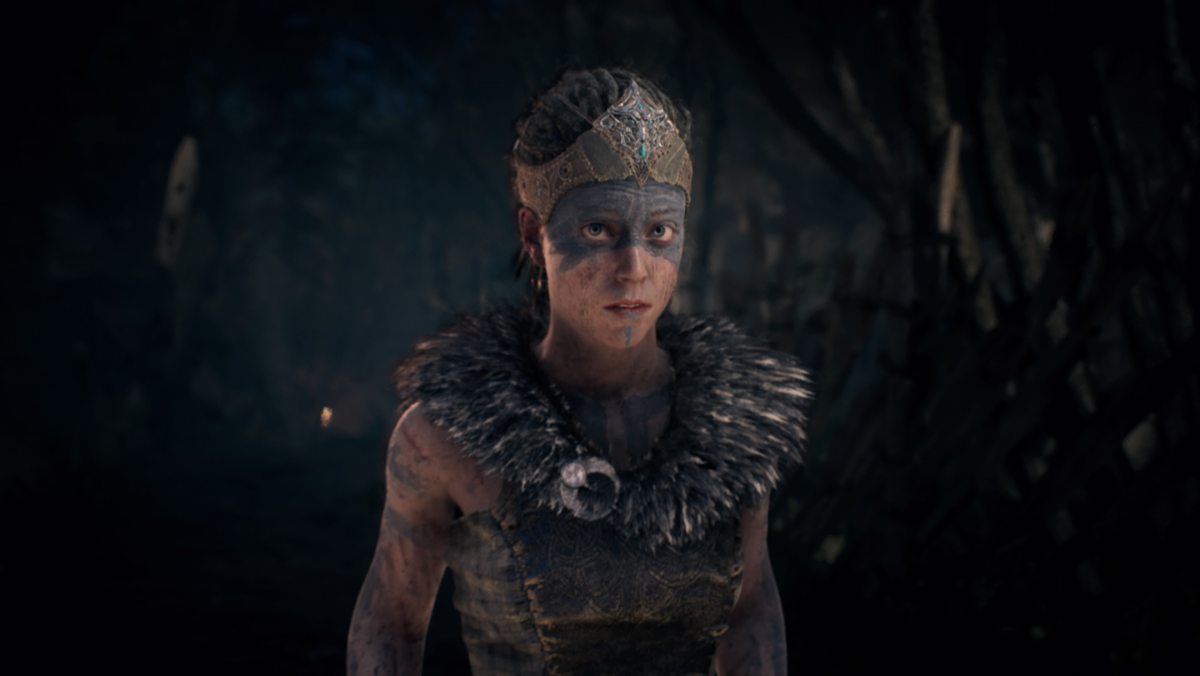
The player-character, Senua, in the game Hellblade (screenshot).

**Figure 3:**
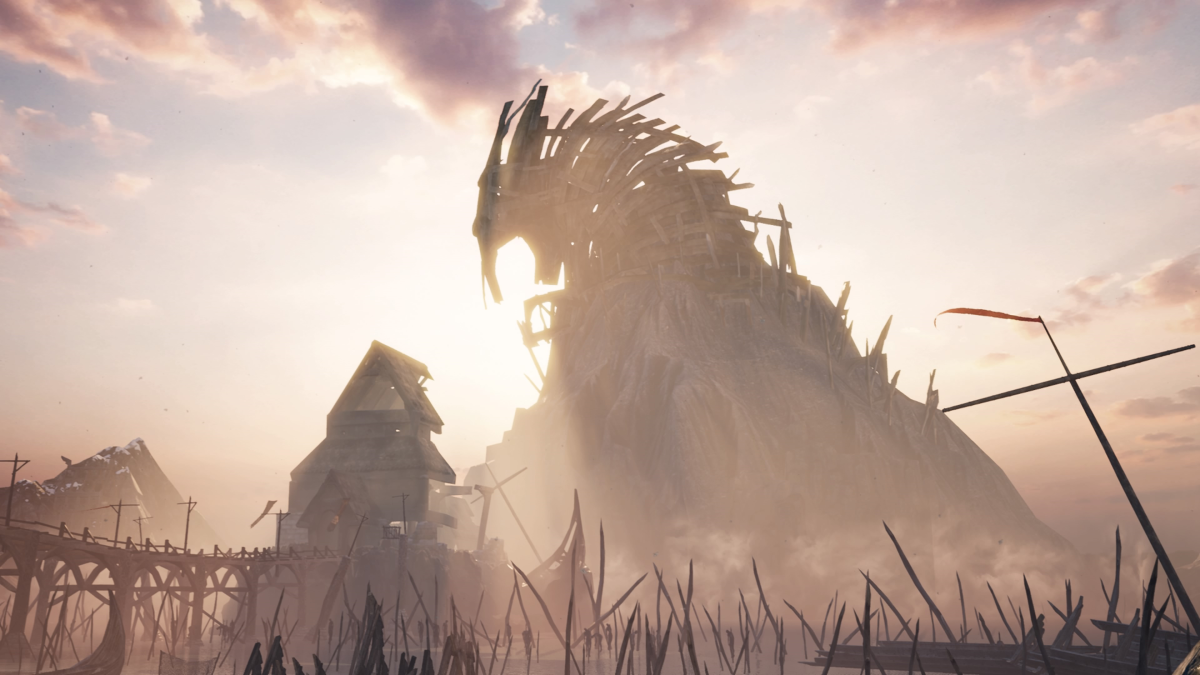
Hellblade's representation of the Norse mythological land of Helheim (screenshot).

**Figure 4:**
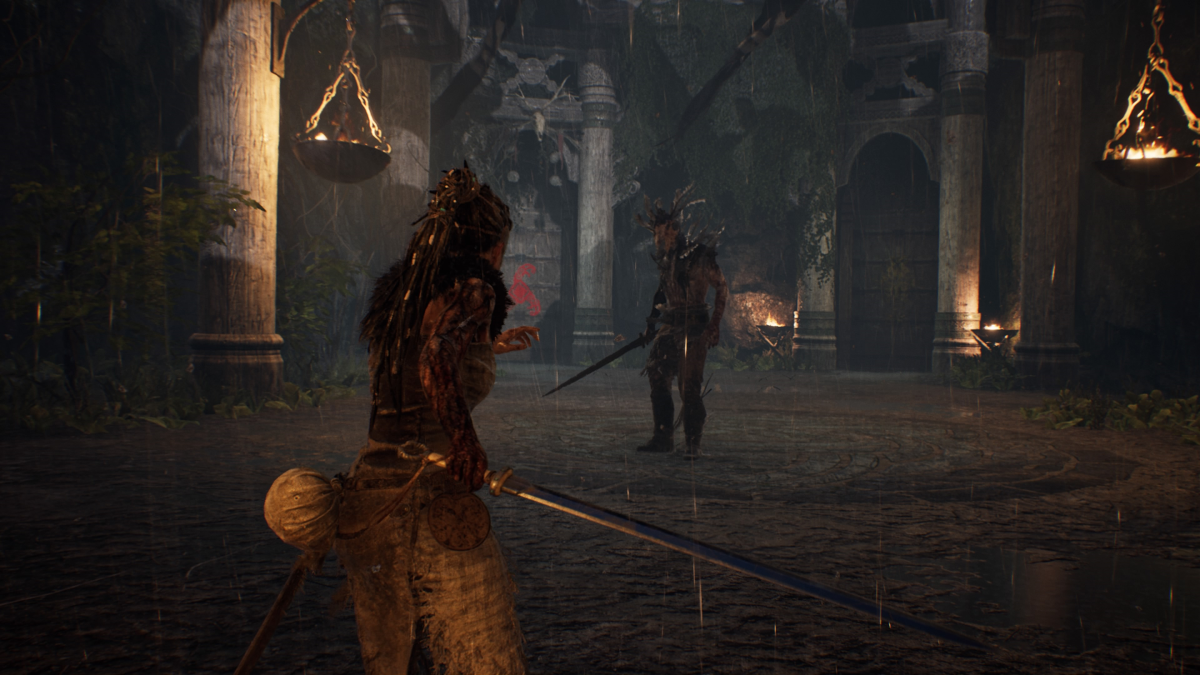
Senua preparing to fight against Norsemen in the game Hellblade (screenshot).

In Hellblade, players are given control of Senua, a female Pict warrior struggling with psychosis after her village is destroyed and the love of her life is killed (see Figure 2). The game centers on Senua’s perceived journey through the Norse mythological land of Helheim in order to save her love from the Norse goddess of death, Hela (see Figure 3). Throughout the game, Senua fights her way through a number of seemingly demonic Norse warriors and mythological creatures while also dealing with various symptoms of her illness, such as auditory and visual hallucinations (see Figure 4). Through the game’s use of storytelling and narrative, Hellblade presents a novel and engaging depiction of psychosis that is crafted within the game’s mechanics.

Hellblade is a game rooted in a time and historical setting that frames Senua’s mental health disorder as a curse. Through visions and stories, players learn that much of the tension in Senua’s life arises from the stigma attached to societal reactions to her “curse” rather than the disorder itself [46]. This stigma includes an abusive relationship with her father and imposed isolation from her community. These past traumas are frequently touched upon with relation to how Senua deals with her disorder, her memory of her mother who also suffered from similar symptoms, and her own role in society. Senua’s father acts as one of the many voices within Senua’s mind as she progresses in the game, consistently demeaning her choices and abilities. These themes of isolation and stigma speak to the ever-present nature of these societal misunderstandings for those who deal with mental illnesses today.

Hellblade’s story, though largely informed by Senua’s psychosis, includes a larger narrative built upon Senua’s journey through Norse mythology. These depictions of Norsemen and their lands are shaped by Senua’s own understandings of Norse mythology told to her by a mysterious man known as Druth who shared these stories with her while she had been exiled from her village. As such, players see horrific representations of Norse warriors attempting to stop Senua during her journey. These monstrous enemies provide more intimidating enemies for players to deal with while also flipping the common depiction of those with mental illness as dangerous or homicidal back onto those around Senua. This dual narrative of traversing Norse mythology while also discovering Senua’s own past and internal strife is important because it provides Hellblade with an engaging setting. Unlike other serious games that explicitly focus on a disorder, psychosis is instead purely a part of the gameplay, experienced through the game’s mechanics. Rather than a game about psychosis, Hellblade is an attempt to create a game that allows the player to experience symptoms similar to psychosis.

This interplay between creating a game and respectfully presenting psychosis was essential to Hellblade’s success. As mentioned before, depictions of more serious topics in games are typically met with accusations of trivialization or further propagating stigmatized stereotypes [47]. That being said, it is also important to ensure that games pertaining to serious topics such as mental health are approachable. For instance, the inclusion of Norse mythology and demonic Viking enemies helps promote an upkeying of Hellblade to the ludic frame, a play experience. Various scenes or interactions within the game, such as Senua’s pleading for her auditory hallucinations to stop, can lead to a downkeying of the play experience as players begin to frame the experience as serious or simply no longer ludic. The ability to shift between these frames is important in not only keeping the player actively engaged with the larger narrative of Senua’s quest to Helheim, but also allows the developers to bring more serious, empathetic moments.

One of the more interesting aspects of the overall story Hellblade provides is the fact that Senua’s reality is never fully questioned [46]. In a game rife with questions of sanity and what is or is not real, Senua’s journey is never placed in relation to “true reality.” Much like those who deal with these hallucinations, Hellblade leaves this question of reality solely for the player to decide. Throughout interviews and diaries, Antoniades argues that reality itself is nothing more than our own interpretation, thus there is no reason to ever relate Senua’s journey to some other point of reference [38]. Players are meant to experience Senua’s reality, as it is wholly real to her. By the end of the game, it is unclear what aspects of Senua’s tale are a part of her psychosis versus reality. By taking this approach, Ninja Theory continues to validate Senua’s experience and reinforces her perspective, rather than removing the veil and re-establishing the player as the driving narrative force [12].

### The Mechanics of Mental Illness

**Figure 5:**
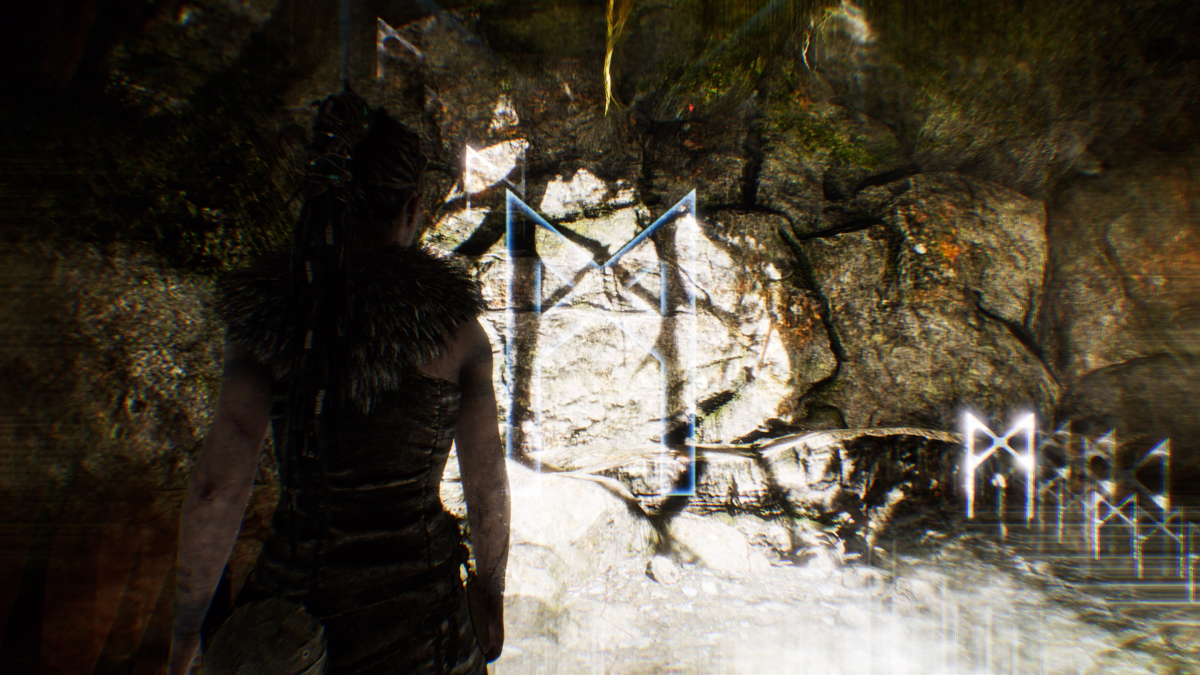
Example of a pareidolia pattern puzzle in the game Hellblade (screenshot).

Throughout the game, numerous game mechanics illustrate a variety of possible symptoms of those suffering from psychosis. Visual and narrative twists are frequently presented through the use of hallucinations and altered realities. Furthermore, various environmental puzzles rely on visual hallucinations and notions of pareidolia, the finding and forming of seemingly insignificant patterns in the everyday (see Figure 5).

Unlike most combat-oriented games, Hellblade includes no extra visual information for the player on the screen, which is referred to as a heads-up display. There is no health meter, no compass to help with direction, and no real tutorial to teach the player combat. In fact, combat within the game is largely repetitive, persisting of 1-2 buttons pressed repeatedly to perform combinations. This is likely because the true goal of Hellblade is not to fight your way through the various Norse enemies present in the game, but to truly engage with Senua’s story. De-emphasizing combat once again helps to frame the experience of Hellblade toward a more serious presentation of psychosis.

**Figure 6:**
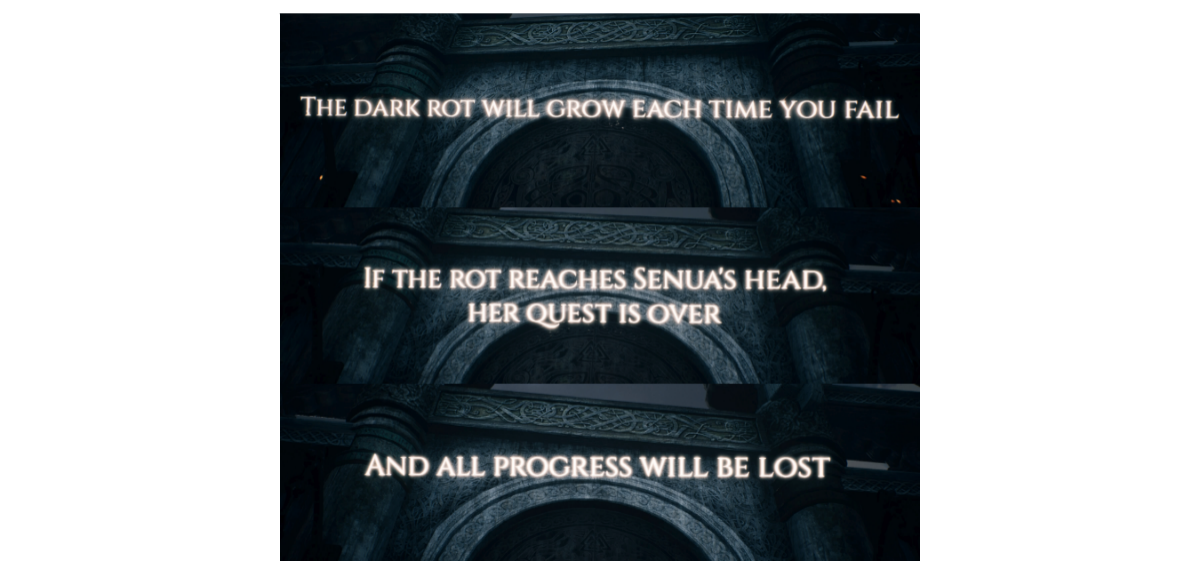
Sequence of permadeath warnings in the game Hellblade (screenshot).

The inclusion of permadeath, a game mechanism in which players lose all progress within the game after failing to complete it within a given number of tries, is introduced early in the playthrough of Hellblade and was one of the most controversial topics after the game was released (see Figure 6). While this turned out to be a bluff intended to promote feelings of dread and anxiety for players akin to those suffering with various mental health disorders, the sheer possibility of a permadeath mechanic within the game was viewed by some as a heavy-handed metaphor for a full break from sanity or suicidal machinations [43]. This sort of response suggests that the rumored inclusion of a permadeath mechanic acted as an upkeying or loosening of Hellblade’s depiction of psychosis, which some may consider inappropriate.

### The Technology of Mental Illness

These initial efforts to address the playful nature of digital games and the ludic frame are important in setting Ninja Theory’s desired tone of Hellblade for the player. Yet, the design choices inherent within Hellblade also work to further downkey the ludic nature of the game. Ninja Theory’s focus is on providing an immersive experience with a level of technological detail rarely seen in games to draw the player toward identifying with Senua. Senua’s struggle is brought to life via the acting of Melina Juergens and the use of both 3D motion capture and facial camera rigs. These were used in an attempt to capture every minor change in facial expression, while also providing harrowing levels of character detail as she experiences the anguishes associated with her journey [47]. This level of graphical fidelity also allowed the designers to experiment with various forms of environmental changes and camera tricks to replicate hallucinations and visual irregularities reported in their interviews with those dealing with mental illness [47].

The most notable aspect of Hellblade is how it presents sound. Throughout the game, the player hears an ongoing chorus of voices within Senua’s mind. Using binaural 3D microphones, Ninja Theory was able to produce an experience of directional auditory hallucinations that Dr Charles Fernyhough, a professor of psychology investigating auditory hallucinations, argues is one of the best representations of these experiences he has ever used [48]. The use of these various voices pushes the player to understand the internal struggles present for Senua, but their use also acts as a mechanic within the game itself, providing players with information and warnings. These voices are present throughout the game, giving advice, questioning the player’s decisions, and doubting the player’s abilities. These voices address the player directly from the opening scene, which illustrates Senua’s awareness of not only these voices, but seemingly of the player as well. The constant interaction between the voices and the player can be off-putting at first, as players adjust to the sheer amount of information being pushed through their headphones. Research has found that exposing individuals to simulations of hallucinations can increase empathy and understanding in relation to sufferers of mental illness [49,50].

### The Collaboration Process

**Figure 7:**
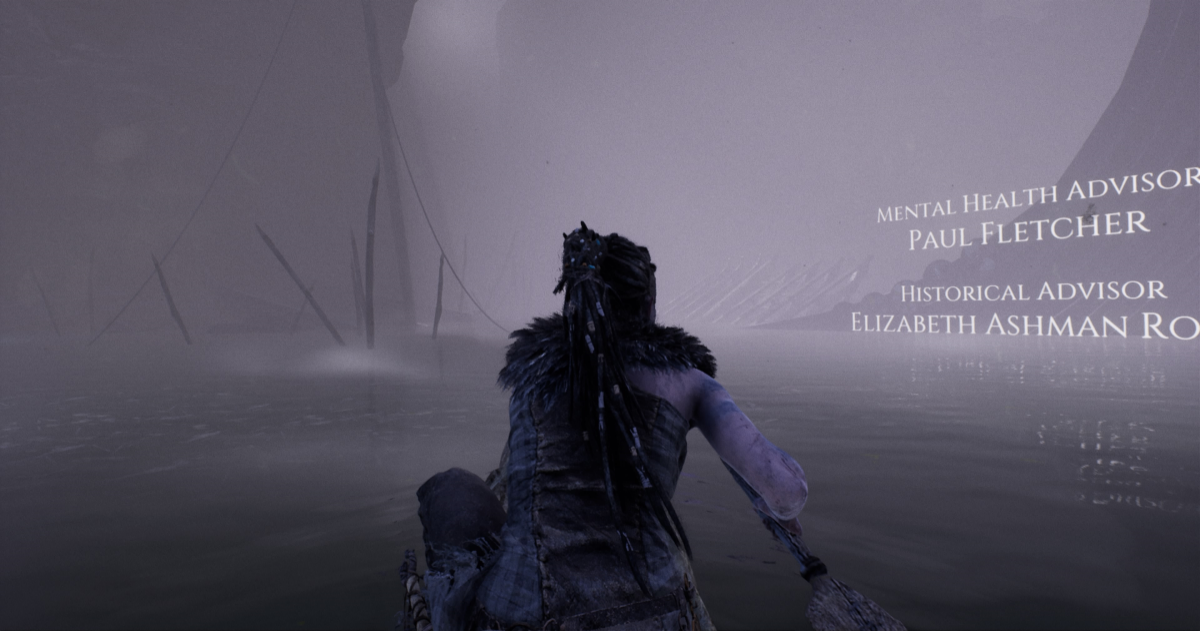
Opening credits of the game, which begin with professional advisors (screenshot).

Throughout the developer diaries and the included documentary on the making of Hellblade, Ninja Theory stresses the role of mental health professionals, scientists, and individuals with mental health problems as advisors throughout the game’s development. In fact, the opening credits of the game begin with credits for both the “mental health advisor” and “historical advisor” positions, rather than the game’s lead designers (see Figure 7). The game’s depiction of Senua’s psychosis was built to reflect conversations, prototyping, and playtesting with these experts to ensure a level of fidelity that is uncommon across most media depictions of mental illness. One fan responded with the following [51]:

I’ve never known how to describe what happens in my mind. You have put words and pictures to how I feel. I showed it to those I’ve been unable to be honest with and connected with the people I love on a level I never have before.

From the initial decision to include a depiction of mental health within Hellblade, Ninja Theory reached out to researchers, mental health professionals, and those who were currently dealing with various symptoms of mental health disorders to craft a genuine representation within their game. This devotion to collaboration is evident within the game’s opening scene, as the very first person credited is Dr Paul Fletcher, a psychiatrist and professor of health neuroscience at the University of Cambridge. Dr Fletcher’s expertise was sought-after early in the design process and continued throughout development [38].

Dr Fletcher’s own work with psychosis served as a source for Hellblade’s focus on pattern-seeking behaviors and themes of isolation and stigma in Senua’s past. This connection with Dr Fletcher along with the support of Wellcome Trust, a charitable organization dedicated to supporting projects devoted to improving global health, allowed Ninja Theory to collaborate further with other mental health professionals and service users via interviews and collaborative prototyping [47]. One key resource was Recovery College East, which is located in England. This is a small group of mental health practitioners and people dealing with mental health problems who willingly shared their own experiences, particularly various forms of visual hallucinations, and helped to playtest certain aspects of the game [41]. For audio hallucinations, a number of other experts, such as Dr Charles Fernyhough, were consulted. These consultations typically involved readings, research talks, playtesting, and prototyping [48]. In this way, Ninja Theory ensured that their game design decisions were being seen and revised based on experts’ knowledge in these fields.

A common thread throughout every interview with experts during their work with Hellblade spoke to the problems associated with stigmatization of these symptoms and illnesses [47]. In response to Hellblade, one fan spoke to the game’s impact [51]:

I had a psychotic break several years ago, my brother never understood. I overheard him say he was ashamed of me. After this game, he turned to me and said he was sorry. You got a message across that I never could.

The chance to collaborate with game developers on a project related to mental health was met with hopes of helping alleviate these stigmas. This level of research and expertise is particularly uncommon within commercially driven game design, yet helps to illustrate lead designer Tameem Antoniades’ devotion to a respectful presentation of Senua’s mental illness. This devotion is contextualized even further through the inclusion of a 25-minute video detailing the various research and resources Ninja Theory used to depict psychosis as well as resources for more information for those interested in learning more [38]. Ultimately, the inclusion of these contextual elements outside of the game itself work to change the frame of the gameplay experience toward one that is serious, respectful, and educational.

**Figure 8:**
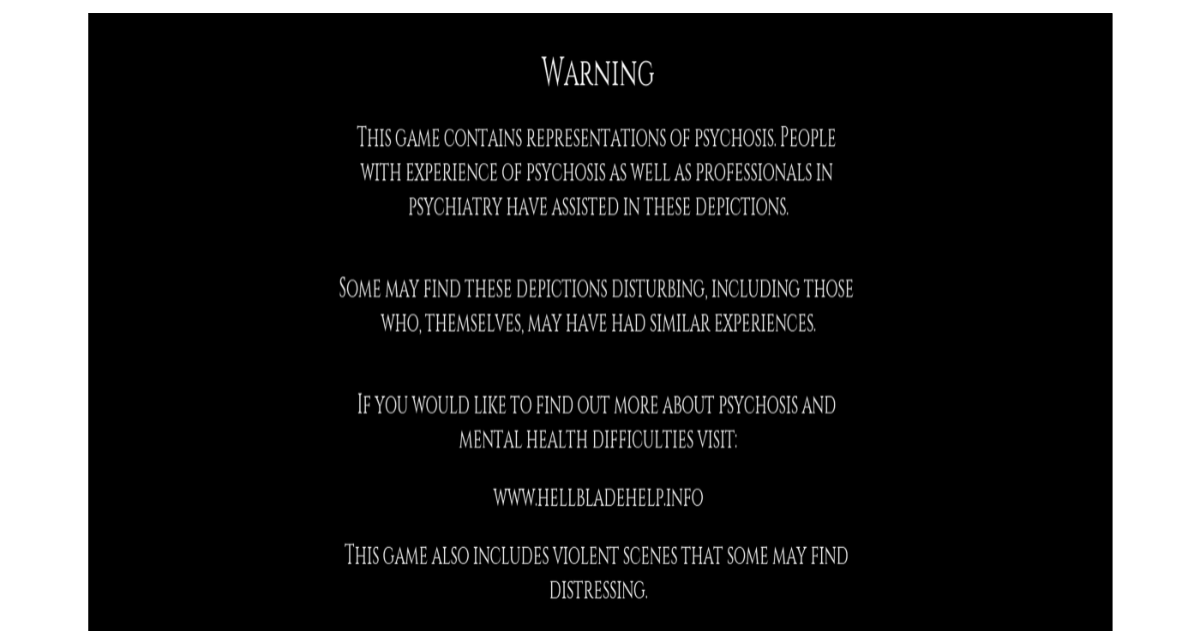
Opening warning screen for the game Hellblade (screenshot).

In an interview with PC Gamer, Ninja Theory’s founder Tameem Antoniades noted the stress and sense of obligation he felt in attempting to successfully and respectfully depict symptoms of mental illness in a meaningful way [52]. Similar to Chapman and Linderoth’s [8] educational framing, Hellblade is presented as a vehicle for awareness and destigmatizing mental illness. Hellblade and Ninja Theory’s websites include various resources for players to learn more about mental illness and potential places to seek help. When loading the game, players are met with an initial warning message that explains the game’s focus on psychosis, the use of experts to provide a faithful representation, and resources for anyone who wants to learn more (see Figure 8). By producing this information before a player can even reach the main menu, Ninja Theory is attempting to immediately downkey from the ludic frame of playing a game to emphasize the seriousness of the topic.

Even before the game’s release, Ninja Theory’s willingness to share their design experience through these developer diaries emphasized their hopes for Hellblade to respectfully present the realities of mental illness. These developmental diaries began as a chronicle and guide for other small teams of developers to independently create and publish a major commercial game. Given outside interest in Hellblade’s depiction of mental illness throughout the game’s development, these diaries and interviews also shifted toward a primer for how game developers can approach serious topics through collaboration with experts, prototyping with caretakers and those who have lived experience with these illnesses, and designing game mechanics that make these issues tangible.

## Discussion

### Principal Findings

In Ninja Theory’s final developmental diary, the creators of Hellblade admitted to a growing sense of fear as they approached the game’s release. Tameem Antoinades, the lead developer, and Melina Juergens, the actress for Senua, both openly admit dreading the idea that the game may, in some way, offend those dealing with psychosis or other forms of mental illness [41]. While much of the reception for Hellblade was positive, including five awards from the BAFTA Game Awards, it did not escape criticism for its use of mental illness within a game setting. Some commentators posited that Hellblade may have overstepped by gamifying aspects of psychosis, appropriating mental illness for commercial use, and even reinforcing myths about mental illness [43].

In this paper, we have argued that it is essential that we examine the design practices that encourage an increased empathetic understanding of people experiencing mental health disorders. Furthermore, we also argue that we must examine the design decisions that allow for a game to effectively represent mental illness within a larger-scale project, rather than a short game designed solely for intervention. In order to do so, we conducted a case study that analyzes Hellblade and discusses the design processes of Ninja Theory. Hellblade is important because it is perhaps the best-reviewed and best-selling video game to attempt a well-researched and appropriate representation of mental health through the medium of digital games [13]. The results of this exploratory study shed light on how design practices can build upon collaboration between researchers and developers to present a successful and potentially helpful depiction of mental disorders.

Our analysis indicates that games should continue to approach depictions of mental health through collaborative design and prototyping with experts. In particular, researchers and game designers should begin working together from the earliest stages of development. Game design should be informed by a strong body of research when dealing with more serious topics, as Ninja Theory demonstrated by promptly seeking Dr Paul Fletcher’s consultation. Perhaps more importantly, for the sake of serious games, researchers and practitioners must take an active role in game design as early as possible. This allows for innovation in design that can provide new forms of embodied experiences, which may help to promote empathy and awareness of mental health issues [51,52]. One perceived benefit of games with relation to mental health interventions is the ability to present new ways of engaging with users [17], but poor game design choices will usually result in poor outcomes [20]. Researchers must be willing to develop or work with game designers to create games that can not only successfully approach mental health topics, but also capitalize on the advantages inherent in using digital games. Knowledge of how games and their mechanics can be understood from both the ludic frame and possibly rekeyed toward a more referential understanding, as noted in our analysis of Hellblade, can facilitate a successful reception of a commercial video game that addresses mental health.

First, transparency is essential. Ninja Theory was very transparent regarding their intentions to make an accurate and empathetic portrayal of mental illness via their development diaries, documentaries, and the warning screen that welcomed new players. Second, input from key stakeholders is also essential. Ninja Theory’s consultation of mental health professionals, scientists, and those with various mental illnesses throughout the development of Hellblade resulted in an accuracy and educational legitimacy rarely seen in mainstream video games.

Finally, technological accuracy that leverages the affordances of video games can help convey mental illness in new and effective ways. Ninja Theory’s use of technologies, such as motion capture and binaural sound recordings in an attempt to provide an empathetic experience similar to the lived experience of those suffering with mental illness, is needed to help destigmatize mental health. These downkeying design decisions culminated in a game that was well-received not only by reviewers, but also the gaming public, going on to sell millions of copies while sparking new conversations and understanding around mental health.

While Hellblade is one of a select few commercial games to put so much reliance on collaboration with researchers and professional advisors, serious game design relies on developers and researchers working together to create a beneficial experience [53]. As digital games have grown with technology and culture acceptance, the ability to portray more realistic content has resulted in an increased desire for fidelity in games. Researchers and professional advisors as collaborators have become common for games that are providing increasingly more realistic depictions of the world around them [54]. Although it is unlikely that large-scale game development will have a major shift toward better-informed, less stereotypical depictions of serious topics, Ninja Theory has shown that more conscientious efforts can be made in the AAA game development space and be successful. As the technological affordances of games continues to grow and games challenge notions of play, experience, and art, developers will need expert advisors to create these experiences. Likewise, willing mental health practitioners and researchers should be open to consult on these larger, commercial projects to ensure a more sincere, potentially empathetic depiction of those suffering from mental ill-health.

### Limitations

Though this study provides an important first step toward understanding the design and impact of one of the more interesting cases of digital games addressing mental health, it is not without several limitations. First, this work is largely exploratory. Future studies may wish to more systematically examine the impact of Hellblade via online forums such as Reddit or Discord. Second, while Hellblade provides an incredibly important case study for both game designers and psychological practitioners, it is still a single case study. Future studies may wish to conduct a frame analysis and community-impact assessment via more case studies to better understand how the framing of games helps benefit games’ attempts to overcome the stigma associated with depictions of more serious topics.

### Conclusions

Hellblade provides numerous avenues for interesting research questions. This paper argues that the framing and design decisions in Hellblade allow it to successfully tackle the potentially troubling topic of mental illness in a video game. For us to adequately explore the capabilities of commercial games that deal with controversial topics, we must examine not only the games themselves, but the design processes behind them. Our hope is that this paper will act as a stepping stone for further research in two ways: first, by illustrating the value of a single game case study, from design to reception, and second, to inform future research possibilities related to games dealing with serious topics such as Hellblade.

## Abbreviations

BAFTA: British Academy of Film and Television Arts
